# Distinct pituitary hormone levels of 184 Chinese children and adolescents with multiple pituitary hormone deficiency: a single-centre study

**DOI:** 10.1186/s12887-019-1819-6

**Published:** 2019-11-14

**Authors:** Fengxue Wang, Jinyan Han, Xiaohong Shang, Guimei Li

**Affiliations:** Department of Pediatrics, Shandong Provincial Hospital Affiliated to Shandong University, 9677 Jingshi Road, Jinan, 250014 Shandong China

**Keywords:** Pituitary hormone levels, Children, Adolescents, Multiple pituitary hormone deficiency

## Abstract

**Background:**

Pituitary tumors and/or their treatment are associated with multiple pituitary hormone deficiency (MPHD) in adults, but the distinct pituitary hormone profile of MPHD in Chinese children and adolescents remains unclear.

**Methods:**

Patients with MPHD were divided into four groups according to their MRI results: 1) pituitary stalk interruption syndrome (PSIS); 2) hypoplasia; 3) normal; and 4) tumor survivor.

**Results:**

Among the 184 patients, 93 patients (50.5%) were with PSIS, 24 (13.0%) had hypoplastic pituitary gland, 10 (5.4%) patients were normal, and 57 (31.0%) were tumor survivors. There was an association between abnormal fetal position and PSIS (*P* ≤ 0.001). The CA/BA in PSIS, hypoplasia, normal, tumor survivor groups were 2.27 ± 1.05, 1.48 ± 0.39, 1.38 ± 0.57, 1.49 ± 0.33, and HtSDS were − 3.94 ± 1.39, − 2.89 ± 1.09, − 2.50 ± 1.05, − 1.38 ± 1.63. Patients in PSIS group had the largest CA/BA (*P* ≤ 0.001 vs. hypoplasia group, *P* = 0.009 vs. normal group, *P* ≤ 0.001 vs. tumor survivors) and lowest HtSDS (*P* ≤ 0.001 vs. hypoplasia group, *P* = 0.003 vs. normal group, *P* ≤ 0.001 vs. tumor survivors). The levels of TSH in the PSIS, hypoplasia, normal, and tumor survivor groups were 1.03 ± 1.08 (*P* = 0.149 vs. tumor survivors), 1.38 ± 1.47 (*P* = 0.045 vs. tumor survivors), 2.49 ± 1.53 (*P* < 0.001 vs. tumor survivors), and 0.76 ± 1.15 μIU/ml. The levels of GH peak in PSIS, hypoplasia, normal, tumor survivor groups were 1.37 ± 1.78, 1.27 ± 1.52, 3.36 ± 1.79, 0.53 ± 0.52 ng/ml and ACTH were 27.50 ± 20.72, 25.05 ± 14.64, 34.61 ± 59.35, 7.19 ± 8.63 ng/ml. Tumor survivors had the lowest levels of GH peak (*P* ≤ 0.001 vs. PSIS group, *P* = 0.002 vs. hypoplasia group, *P* ≤ 0.001 vs. normal group) and ACTH (all the *P* ≤ 0.001 vs. the other three groups).

**Conclusion:**

The frequency of PSIS is high among children and adolescents with MPHD. The severity of hormone deficiencies in patients with MPHD was more important in the tumor survivor group compared with the other groups.

## Background

Multiple pituitary hormone deficiency (MPHD) is a type of chronic, life-long hypopituitarism and refers to the impaired production of one or more anterior pituitary hormones in addition to growth hormone (GH) [1]. Hypopituitarism may have different etiologies and can be acquired or congenital. Pituitary tumors (mostly non-functioning pituitary adenomas) and/or their treatment with pituitary surgery or radiotherapy are the most frequent etiologies of hypopituitarism in adults [2–4]. Other etiologies include non-pituitary lesions and non-cancerous diseases [1]. Regarding the non-cancerous etiologies, about 10–20% of patients show genetic mutations in genes encoding for the transcription factors *LHX3*, *LHX4, PROP1*, and *POU1F1* (*PIT1*) [5, 6]. In the remaining 80–90% of patients, etiologies include perinatal injuries, malformation, trauma, and pituitary stalk dysgenesis [2, 7]. Of note, the incidence of pituitary tumors and lesions in children and adolescents is lower than in adults. Therefore, the etiology of hypopituitarism in children and adolescents may be completely different from adults, and the etiology of MPHD in children and adolescents should be determined and analyzed separately. Besides age, the etiology of MPHD in children and adolescents is less likely to be from past trauma, and more likely to be from birth characteristics.

The pituitary gland is considered the “master gland” for its control over the endocrine glands and physiological processes such as metabolism, growth, and development [8, 9]. Although MPHD may have different etiologies, the severity of hypopituitarism across the different etiologies is unknown. Since hypopituitarism leads to decreased levels of pituitary hormones, the pituitary hormonal levels may provide hints about the severity of MPHD. A magnetic resonance imaging study from Saudi Arabia in 11 children with MPHD revealed that 6/11 children had pituitary hypoplasia, 3/11 had pituitary aplasia, and 2/11 had normal MRI [10]; unfortunately, they did not examine the hormone levels. An early study of 15 children with MPHD showed that head MRI abnormalities were common and associated with peak GH levels < 3 μg/L [11]. A study of 33 children with MPHD showed abnormal pituitary stalk in 75.7% and decreased pituitary hormones in all 33 patients [12]. Two studies showed that the absence of the pituitary stalk was associated with MPHD and GH deficiency [13, 14]. Kandemir et al. [15] showed that the absence of the pituitary stalk was a good indicator of the severity of hormone deficiency.

Although the etiology of hypopituitarism is the focus of numerous papers, there are only a few articles about children and adolescents, and the distinct pituitary hormone profile of MPHD in children and adolescents remains unclear. Therefore, the aim of this study was to analyze the hormone profiles of Chinese children and adolescents with different MPHD etiologies. This study could help pediatricians better understand the distinct hormone levels of MPHD in children and adolescents.

## Methods

### Patients

This was a historical retrospective study of Chinese children and adolescents (2 months to 18 years old) diagnosed with MPHD at the pediatric endocrine outpatient facility of Shandong Provincial Hospital affiliated to Shandong University, from January 2008 (start of the database used to identify the patients) to January 2018. The exclusion criteria included a history of dementia, encephalitis, stroke, other neurological diseases, schizophrenia, depression, and other psychiatric diseases. Patients or their parents or legal guardians provided verbal consent for their non-identifiable data to be collected and analyzed. The Medical Ethics Committee of Shandong Provincial Hospital affiliated to Shandong University approved this retrospective data only study with a waiver of informed consent.

### Data collection

Sex, etiology of MPHD, age, height, and weight at the time of MPHD diagnosis, number and type of deficient pituitary hormones, family history, birth information, pituitary hormone levels at the time of MPHD diagnosis, and MRI results were collected.

The diagnosis of MPHD was based on the following criteria: (1) GH deficiency (GHD); (2) concomitant deficiency in one or more pituitary hormones (i.e. thyroid-stimulating hormone (TSH), adrenocorticotropic hormone (ACTH), follicle-stimulating hormone (FSH), luteinizing hormone (LH) and antidiuretic hormone (ADH)); and (3) absence of other diseases that may affect the functioning of the hypothalamus and pituitary gland. Available MRI data was an inclusion criterion. Only patients with a complete data set were included.

The pituitary axis was examined using the following tests. (1) GHD is generally diagnosed in the absence of a significant peak in GH secretion after at least one stimulation test. In this study, the diagnosis was based on GH peak levels < 10 ng/ml following two different GH provocation tests (i.e., the arginine test, 0.5 mg/kg, the maximum dosage was 30 mg; and the levodopa test, 10 mg/kg, the maximum dosage was 500 mg). (2) TSH deficiency was defined as low serum free T4 (FT4 < 12.0 pmol/l) (reference range, 12.0–22.0 pmol/l) with concomitantly normal or decreased serum TSH (reference range, 0.27–4.2 μIU/ml) [16]. (3) Adrenocorticotropic hormone (ACTH) deficiency was assessed by either decreased serum cortisol (COR) level in the morning (COR < 138 nmol/l) or an impaired cortisol serum concentration rise (COR < 550 nmol/l) during insulin-induced hypoglycemia with an inappropriately low serum ACTH concentration. (4) We analyzed patients’ pubertal development at the time of their first visit among patients whose age was above 12-year old. Gonadotropin deficiency was based on the gonadotropin hormone-releasing hormone-stimulation test (triptorelin was administered at 2.5 μg/kg, subcutaneous injection, the maximum dosage was 100 μg, blood was drawn at intervals from 30 min to 2 h after injection, and the cut-off point of blunted response was 2.8 mIU/ml for luteinizing hormone (LH) and/or 3.7 mIU/ml for follicle-stimulating hormone (FSH)), or basal levels of FSH and LH below the assay sensitivity (0.1 mIU/ml) on the basis of delayed or absent pubertal development. (5) ADH is unstable and difficult to detect. Central diabetes insipidus (CDI) can reflect ADH deficiency. The diagnosis of CDI was based on confirmed hypotonic polyuria. The fluid deprivation-vasopressin test was carried out for confirmation. All patients underwent the same testing protocol, and all tests were carried out after overnight fasting.

### Height and weight

Height was measured in the morning, by the same medical team, and expressed in cm. Height measurements were standardized to age and sex and were expressed as standard deviation scores (SDS) relative to the chronological age (CA), according to the Growth Charts for Chinese Children and Adolescents (2009) [17, 18]. Genetic target height (THt) was calculated according to the following formula: ([height of the father + height of the mother]/2)-6.5 cm for girls and + 6.5 cm for boys. THt measurements were also standardized to age and sex and were expressed as standard deviation scores (SDS), according to the Growth Charts for Chinese Children and Adolescents (2009) [17, 18]. Weight was measured in the morning, in the fasting state, at every visit, and expressed in kg. Body mass index (BMI) was calculated as weight (kg) divided by height squared (meters). BMI values were transformed into BMISDS, based on the Normative Values for Chinese Children and Adolescents (2009) [19], in order to minimize the confounding effects of age and sex.

### Imaging

**Fig. 1:**
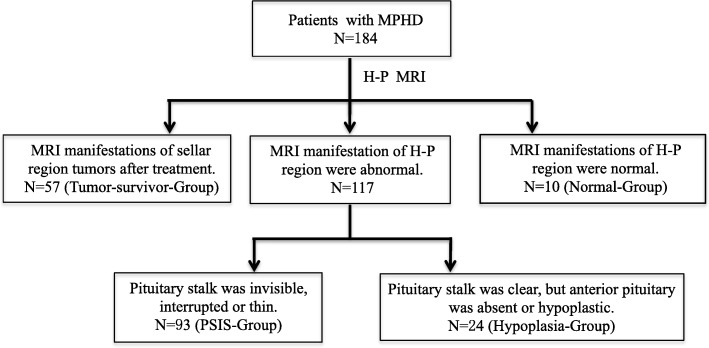
The flow chart

Bone age (BA) was determined by a single-blinded observer using a left hand-wrist radiograph according to Greulich and Pyle’s standards. Hypothalamus-pituitary MRI (H-P MRI) was performed using a 3.0 T scanner (Siemens, Erlangen, Germany) in the sagittal and coronal planes on T1- and T2-weighted images. Gadolinium diethylenetriamine pentaaceticacid (Gd-DTPA) was administered at 0.1 mmol/kg. The slice thickness was 3 mm. The height of the non-tumor pituitary gland was measured. All images were reviewed by at least two experienced radiologists and evaluated for central nervous system malformations with specific attention to the height of anterior pituitary, the visibility of the pituitary stalk and the location of ectopic posterior pituitary. A full agreement had to be reached on the positive nature of MRI findings. The diagnosis of pituitary stalk interruption, pituitary hypoplasia or normal pituitary was decided by the same experienced radiologist. Based on their judgments, the patients were deemed to have invisible (no visible pituitary stalk was seen in the region between the hyperintense signal of ectopic posterior pituitary and anterior pituitary), interrupted, or thin pituitary stalk and they were diagnosed with pituitary stalk interruption syndrome (PSIS). Patients with clear pituitary stalk, but absent or hypoplastic anterior pituitary gland were diagnosed with pituitary hypoplasia. Patients with confirmed tumor pathology or diagnostic certificate issued by a neurosurgeon were considered tumor survivors. H-P MRI was performed after tumor treatment. Thus, the patients were divided into four groups: PSIS, hypoplasia, normal MRI, and tumor survivors. Figure 1 presents the grouping information.

### Statistical analysis

Collected data were analyzed using IBM SPSS Statistics 25. In this study, the independent variable was the MRI grouping, and the hormone levels were the dependent variables. The continuous data were tested for normal distribution using the Kolmogorov-Smirnov test and were all found to be normally distributed. The descriptive statistics of the quantitative variables were presented as means ± standard deviations (SD) and in percentages (%). Groups were compared using the Student’s t-test, Kruskal-Wallis test, and Mann-Whitney U test, depending on data type. The chi-square test was used to analyze the relevance between birth information and PSIS. The threshold for statistical significance was set at 0.05.

## Results

### Characteristics of the patients

Among the 184 patients, 137 (74.5%) were male, and 47 (25.5%) were female. The different characteristics of patients with MPHD in the different groups are shown in Table 1. Among all patients, 93 patients (50.5%) were with PSIS, 24 (13.0%) had hypoplastic pituitary gland, 10 (5.4%) patients were normal, and 57 (31.0%) were tumor survivors. In the tumor survivor group, 48 (48/57) children and adolescents had craniopharyngioma (CP) and were treated by surgery or combined with radiotherapy, four (4/57) had intracranial germinoma, three (3/57) had pituitary adenomas, one (1/57) had Langerhans cell histiocytosis, and one (1/57) had Rathke’s cleft cyst.

**Table 1:** The characteristics of patients with MPHD in the different groups

	PSIS (*n* = 93)	Hypoplasia (*n* = 24)	Normal (*n* = 10)	Tumor survivor (*n* = 57)	P1	P2	P3	P4	P5	*P6*
N (Males/females)	73/20	19/5	7/3	38/19						
THt SDS	−0.31 ± 0.76	−0.39 ± 0.66	−0.40 + ±0.39	− 0.26 ± 0.63	0.638	0.714	0.678	0.964	0.406	0.500
CA (years)	9.64 ± 5.04	10.40 ± 4.78	10.16 ± 4.76	8.90 ± 3.70	0.507	0.756	0.233	0.895	0.083	0.263
BA (years)	5.83 ± 4.08	7.62 ± 3.68	9.33 ± 3.69	6.44 ± 3.30	0.053	0.011	0.342	0.226	0.160	0.015
CA/BA	2.27 ± 1.05	1.48 ± 0.39	1.38 ± 0.57	1.49 ± 0.33	≤0.001	0.009	≤0.001	0.557	0.906	0.409
HtSDS	−3.94 ± 1.39	−2.89 ± 1.09	−2.50 ± 1.05	−1.38 ± 1.63	≤0.001	0.003	≤0.001	0.363	≤0.001	0.042
BMISDS	0.16 ± 1.38	0.39 ± 1.34	−0.72 ± 1.74	0.92 ± 1.36	0.468	0.066	≤0.001	0.052	0.111	0.001
Anoxia or asphyxia at birth	61	8	2	0						
Breech/Foot presentation	46	4	1	0						
Head presentation	28	15	5	35						
Cesarean section	19	5	4	22						
Pituitary Height (mm)	2.59 ± 0.87	2.24 ± 0.87	4.80 ± 1.68	/^a^	0.083	≤0.001	/	≤0.001	/	/

P1: P-value between PSIS-Group and Hypoplasia -Group. P2: P value between PSIS-Group and Normal-Group. P3: P-value between PSIS-Group and Tumor survivor. P4: P value between Hypoplasia-Group and Normal-Group. P5: *P* value between Hypoplasia -Group and Tumor survivor-Group. P6: P value between Normal-Group and Tumor survivor-Group

^a^A large majority of (48/57) tumor survivors were CP survivors, and the normal anatomy of the sellar region was seriously damaged by tumor or the related treatment. So the pituitary height could not be measured in MRI

Seven children and adolescents without a tumor (five males and two females; two with hypoplasia and five with PSIS) underwent gene examination. A mutation in *LHX4* was found in a 16-year old boy with PSIS. A mutation in *GH1* was found in a 13-year old boy with hypoplasia.

The four groups had similar THt SDS (all *P* > 0.05). BA of the tumor survivor group was younger than that of the normal group (*P* = 0.015). All patients had delayed BA. CA/BA of the PSIS group was higher than that of the hypoplasia, normal, and tumor survivor groups (*P* ≤ 0.001, *P* = 0.009 and *P* ≤ 0.001, respectively). The height SDS of the PSIS group was lower than that of the hypoplasia, normal, and tumor survivor groups (*P* ≤ 0.001, *P* = 0.003, and *P* ≤ 0.001). The children and adolescents in the normal group had higher pituitary height than the PSIS and hypoplasia groups (*P* ≤ 0.001 and *P* ≤ 0.001, respectively). There were associations between PSIS and abnormal fetal position and anoxia/asphyxia (*P* ≤ 0.001).

### Hormone levels

**Fig. 2:**
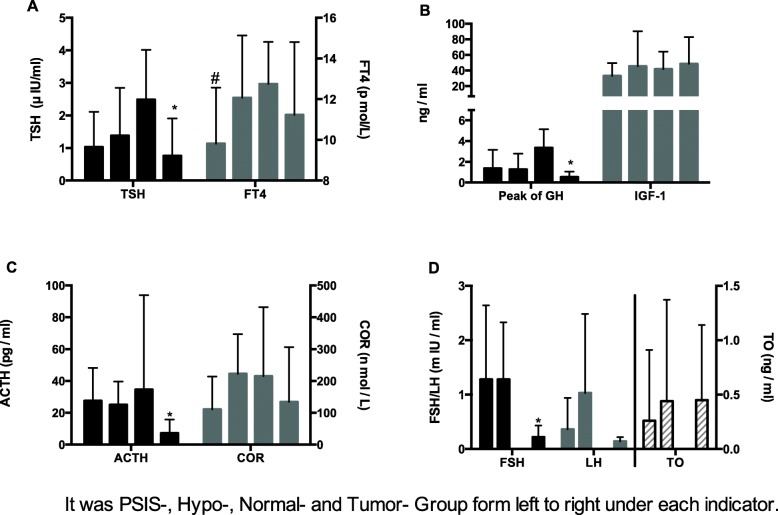
Pituitary hormonal levels of MPHD patients in different groups. **a** * Level of TSH in Tumor-Group was significantly lower than Hypo-Group and Normal-Group (*P* = 0.011 and *P* ≤ 0.001, respectively). #FT4 level of patients in PSIS-Group was significantly lower than Hypo-, Normal- and Tumor- Group (*P* ≤ 0.001, *P* = 0.005 and *P* = 0.002, respectively). **b** *The GH peak of Tumor-Group was significantly lower than that of PSIS, Hypo- and Normal groups (all the *P* ≤ 0.001). **c** *The level of ACTH of Tumor-Group was also significantly lower than the other three groups (all *P* ≤ 0.001). **d** *The level of FSH in Tumor-Group was significantly lower than PSIS-Group and Hypo-Group (*P* = 0.020 and *P* = 0.006, respectively)

Table 2 and Fig. 2 present the hormone levels. The levels of TSH in the tumor survivor group were significantly lower than in the hypoplasia and normal groups (*P* = 0.045 and *P* ≤ 0.001, respectively). FT4 levels of patients in the PSIS group were significantly lower than in the hypoplasia, normal, and tumor survivor groups (*P* ≤ 0.001, *P* = 0.002 and *P* = 0.008, respectively), but there were no difference among the hypoplasia, normal, and tumor survivor groups. The GH peak of the tumor survivor group was significantly lower than that of the PSIS, hypoplasia, and normal groups (*P* ≤ 0.001, *P* = 0.002, and *P* ≤ 0.001). The GH peak of the normal group was higher than in the PSIS and hypoplasia groups (*P* ≤ 0.001 and *P* = 0.002). The ACTH levels of the tumor survivor group were significantly lower than in the other three groups (all *P* ≤ 0.001). There was no difference among the PSIS, hypoplasia, and normal groups (*P* > 0.05). The level of FSH in the tumor survivor group was significantly lower than in the PSIS and hypoplasia groups (*P* = 0.019 and *P* = 0.006, respectively), but there was no such difference on LH and testosterone levels. The LH level of patients in PSIS group was higher than that of the hypoplasia group (*P* = 0.015). No comparison could be made among the normal group and the other groups due to the small number of patients.

**Table 2:** Pituitary hormone levels of MPHD in different groups

	Normal range	PSIS	Hypoplasia	Normal	Tumor-survivor	P1	P2	P3	P4	P5	P6
FT4	12–22 pmol/L	9.82 ± 2.75	12.31 ± 3.07	12.75 ± 2.07	11.23 ± 3.58	≤0.001	0.002	0.008	0.682	0.200	0.198
TSH	0.27–4.2 μIU/ml	1.03 ± 1.08	1.38 ± 1.47	2.49 ± 1.53	0.76 ± 1.15	0.193	≤0.001	0.149	0.056	0.045	≤0.001
IGF-1	111–555 ng/ml	33.60 ± 16.44	45.49 ± 44.67	41.69 ± 22.29	48.57 ± 34.19	0.039	0.159	≤0.001	0.800	0.737	0.543
GH peak	> 10.00 ng/ml	1.37 ± 1.78	1.27 ± 1.52	3.36 ± 1.79	0.53 ± 0.52	0.801	≤0.001	≤0.001	0.002	0.002	≤0.001
COR	172–497 nmol/l	110.88 ± 103.05	222.73 ± 124.03	215.00 ± 217.30	134.48 ± 171.48	≤0.001	0.009	0.294	0.896	0.025	0.193
ACTH	7.2–63.3 pg/ml	27.50 ± 20.72	25.05 ± 14.64	34.61 ± 59.35	7.19 ± 8.63	0.587	0.423	≤0.001	0.458	≤0.001	≤0.001
FSH peak^a^	mIU/ml	1.28 ± 1.36 ^b^	1.28 ± 1.05^c^	/^d^	0.22 ± 0.21^e^	> 0.99	/	0.019	/	0.006	/
LH peak^a^	mIU/ml	0.36 ± 0.58	1.03 ± 1.45	/	0.14 ± 0.08	0.038	/	0.242	/	0.069	/
T^a^	ng/ml	0.26 ± 0.65	0.44 ± 0.93	/	0.45 ± 0.69	0.507	/	0.429	/	0.979	/

P1: P-value between PSIS-group vs Hypoplasia-group. P2: P-value between PSIS-group vs Normal-group. P3: PSIS-group vs Tumor-survivor-group. P4: *P*-value between Hypoplasia-group VS Normal-group. P5: P-value between Hypoplasia -group VS Tumor-survivor-group. P6: P-value between Normal-group VS Tumor-survivor-group

*PSIS* pituitary stalk interruption syndrome, *FT4* free thyroxine, *TSH* thyroid-stimulating hormone, *IGF-1* insulin growth factor 1, *GH* growth hormone, *COR* cortisol, *ACTH* adrenocorticotropic hormone, *FSH* follicle-stimulation hormone, *LH* luteinizing hormone, *T* testosterone

^a^Patients whose referral age was above 12 years and only analyzed data of male patients, for the number of female patients was small and could not be analyzed

^b^*N* = 33

^c^*N* = 9

^d^*N* = 2

^e^*N* = 10

Frequency of pituitary hormone deficiency in different groups.

Table 3 presents the prevalence of pituitary hormone deficiency in different groups. Among all the patients, 100% patients had GHD, 91.3% (168/184) patients had TSH deficiency, 63.6% (117/184) patients with ACTH deficiency, 28.8% (53/184) patients suffered from CDI. We also analyzed the gonadotropin (FSH/LH) deficiency among the patients above 12 years old at the time of their first treatment (the total number of patients above 12-year was 64), and 49 (49/64) patients had gonadotropin deficiency. The frequency of TSH deficiency was higher than the other pituitary hormones in every group. The frequency of ADH deficiency in non-tumorous patients was lowest. It was noteworthy that the frequency of deficiency of TSH, ACTH, gonadotropin, and ADH in the tumor survivor group was higher than in other groups. Especially the deficiency of ADH was more likely to occur in tumor survivor group.

**Table 3:** Frequency of pituitary hormone deficiency in different groups

Groups	GH N(%)^a^	TSH N(%)^a^	ACTH N(%)^a^	FSH/LH N(n)^b^	ADH N(%)^a^
PSIS-	93 (100%)	86 (92.5%)	56 (60.2%)	32 (38)	9 (9.7%)
Hypo-	24 (100%)	21 (87.5%)	12 (50%)	8 (10)	2 (8.3%)
Normal-	10 (100%)	6 (60.0%)	4 (40.0%)	2 (4)	1 (10.0%)
Tumor-	57 (100%)	55 (96.49%)	45 (78.94%)	7 (12)	41 (71.93%)

^a^N (%):Number of patients with particular hormone deficiency in a particular group (the proportion of cases in the total patients in particular group)

^b^N (n): Number of patients with particular hormone deficiency (number of patients whose referral age was above 12 years at the time of MPHD diagnosed at the first visit)

## Discussion

To date, only a few studies investigated the etiologies of MPHD and the pituitary hormone levels of these patients. In addition, there are even fewer papers about children and adolescents with MPHD. In this retrospective study, we determined the pituitary hormone levels of MPHD in 184 Chinese children and adolescents.

The etiology of MPHD is heterogeneous. MPHD can be familial (i.e., due to gene mutations, of which *PROP1* is the most common) or secondary to diseases that affect the hypothalamic-pituitary system [1, 20]. Pituitary cell destruction by adenomas or associated treatments (e.g., surgery and radiotherapy) accounts for more than 95% of cases of hypopituitarism or MPHD in adults [21], but the incidence of pituitary adenomas is very low in children and adolescents. Therefore, children and adolescents have completely different etiologies of MPHD.

MRI is considered the “gold standard” imaging modality due to its high contrast details, allowing the diagnosis of diseases in the sellar and juxtasellar region [22]. Thus, MRI is especially valuable for the diagnosis of MPHD [23]. In this study, more than 50% (93/184) of the patients with MPHD had PSIS. Only 57 patients (31.0%) had acquired MPHD because of a tumor. Therefore, PSIS may be the most common etiology of MPHD in children and adolescents, which is different from adults [24]. Among all the non-tumor reasons, PSIS was the most common pituitary dysplasia associated with MPHD, as supported by a previous study [25]. More than 50% of the PSIS children and adolescents (61/93) had abnormal fetal positions and anoxia/asphyxia due to difficult labor. These facts may indicate that some relationships among fetal position, anoxia/asphyxia, and PSIS may exist, as supported by a previous study [23].

*LHX4* mutations are heterozygous and can be associated with pituitary stalk interruption [26, 27], as observed in one patient in the present study. A mutation in *GH1* was found in one patient in the hypoplasia group. Gene mutation in *GH1* can lead to IGHD, but this patient was diagnosed with MPHD. A previous study showed that about 5.5% of children with IGHD could develop additional pituitary hormone deficiencies and hypopituitarism is a dynamic condition where new hormone deficiencies may occur as the disease progresses [28]. The positive rate of gene mutation was low in the present study. Genetic etiologies could not be identified in the majority of patients with congenital MPHD despite recent advances. Previous studies showed that genetic abnormalities could be identified in only 5–10% of cases of congenital hypopituitarism [8, 29]. In addition, genetic screening is expensive considering the number of genes that have to be tested.

Previous research showed that the incidence of IGHD in males was higher than in females [29]. In the present study, the number of males with MPHD was higher than females. The exact cause for this difference is unknown, besides the fact that male infants were favored during the implementation of the one-child policy in China [30].

In this study, 31.0% of patients were tumor survivors, the majority of which were CP survivors. CP is the most common brain tumor that can destroy the pituitary function in children [31], which can explain a large number of CP survivors in the tumor survivor group.

In the present study, the diagnosis age was 2 months to 18 years. The large age span of diagnosis may owe to the severity and phenotype of MPHD that could vary due to the different combinations of defective pituitary hormones. The onset of MPHD may be diagnosed very early in the neonatal period and can be noticed in childhood or adolescence or adulthood. In some instances, the full expression of MPHD evolves over time [4]. It is noteworthy that the patients in the tumor survivor group were younger than in the other groups. This may be because of the early onset of brain tumors. In addition, the onset of MPHD is always insidious, and the clinical presentation is always nonspecific [4]. Second, many Chinese parents are familiar with the idea of “delayed puberty”, leading to a higher referral age in the other three groups. Third, a large number of children and adolescents were from rural areas with underdeveloped economy, where little attention is paid to growth and development. All the above reasons contribute to the late referral age of non-tumor children and adolescents. This is particularly important, as the referral age varied inversely with the height SDS.

Some previous studies observed MRI characteristics in patients with MPDH, but few examined the hormone levels in a comprehensive manner [10, 11]. A study of 33 children with MPHD showed abnormal pituitary stalk in 75.7% and decreased pituitary hormones in all 33 patients [12]. Chen et al. [13] and Kornreich et al. [14] showed that the absence of the pituitary stalk was associated with MPHD and GH deficiency. Kandemir et al. [15] showed that the absence of the pituitary stalk was a good indicator of the severity of hormone deficiency. The present study showed that besides GHD, TSH deficiency was the most frequent, followed by ACTH deficiency. In a previous study in adults, FSH/LH deficiency was the most common [24]. The different etiologies, important age differences, and different races may contribute to the discrepancies. According to the present study, the frequencies of TSH, ACTH, and gonadotropin deficiencies in the different groups were different. The reason is unknown since the varying levels of different pituitary hormones in relation to pathological damage or abnormal anatomy still remain unexplained [4]. The frequencies of TSH, ACTH, and gonadotropin deficiencies in the tumor survivor group were higher than in the other groups. Tumor treatment may influence the functions of the sellar region. A large proportion of tumor survivors had CP, and it is acknowledged that surgery with/without radiotherapy represents the standard CP treatment [32]. Almost all patients with CP experience complete panhypopituitarism after tumor resection because hypothalamus and pituitary are damaged and cannot work properly [33].

An important limitation of this study is the lack of genetic assessment results for all patients. The uneven number of male and female patients may also affect the results. The types of tumor treatment might influence the characteristics of the disease, but those characteristics could not be analyzed because of the small numbers of patients in some subgroups. In addition, because of the retrospective nature of the study, tumor data were unavailable for many patients because they were treated at other hospitals. These limitations and the long follow-up with MPHD children and adolescents should be addressed in future studies. Of course, the generalizability of the present study might be limited. Indeed, the results were from a very limited population of patients from a single hospital in China. The genetic variability of this population is thus very limited and cannot be applied to other populations. In addition, biases such as local obstetrical practices, laboratory measurements, and sex imbalance can affect the validity of the results.

## Conclusions

The most common etiology of MPHD in children and adolescents is PSIS, followed by tumors such as CP. The frequency of MPHD may be higher in males than in females. The severity of hormone deficiency in the tumor survivor group is higher compared with the other etiologies.

## Data Availability

All data generated or analyzed during this study were included in this published article.

## Abbreviations

ACTH: Adrenocorticotropic hormone
ADH: Antidiuretic hormone
BA: Bone age
CA: Chronological age
CDI: Central diabetes insipidus
COR: Cortisol
FSH: Follicle-stimulating hormone
FT4: Free T4
GH: Growth hormone
GHD: Growth hormone deficiency
H-P MRI: Hypothalamus-pituitary MRI
LH: Luteinizing hormone
MPHD: Multiple pituitary hormone deficiency
MRI: Magnetic resonance imaging
PSIS: Pituitary stalk interruption syndrome
SDS: Standard deviation scores
THt: Genetic target height
TSH: Thyroid-stimulating hormone
